# Effect of Limbal relaxing incisions during implantable collamer lens surgery

**DOI:** 10.1186/s12886-017-0458-7

**Published:** 2017-05-08

**Authors:** Zhen Li, Yu Han, Budan Hu, Huibin Du, Gengsheng Hao, Xiaoxi Chen

**Affiliations:** Department of Ophthalmology, Leshan People’s Hospital, 635 Wanghaoer Street, Leshan, Sichuan Province 614000 People’s Republic of China

**Keywords:** Corneal astigmatism, Limbal relaxing incisions, Implantable collamer lens, Myopia;phakic intraocular lens, Visual acuity

## Abstract

**Background:**

The limbal relaxing incisions (LRIs) technique is a safe and an inexpensive procedure, which is simple for experts to perform. It can effectively reduce astigmatism and result in a rapid visual rehabilitation. But there are few reports about reducing pre-existing corneal astigmatism by LRI in ICL surgery. Our research was aimed to study the effect of limbal relaxing inci sions during implantable collamer lens (ICL) surgery.

**Methods:**

A prospective analysis reviewing consecutive cases of corneal astigmatism that had either independent ICL surgery (control group) or combined with LRIs (LRIs group). The study population consisted of 45 patients, 85 eyes, with high myopia and regular corneal astigmatism more than 0.50 diopter (D) and less than 3.00 D. The first group received ICL surgery combined with LRIs (limbal relaxing incisions); the control group received only ICL surgery alone. The outcomes considered were uncorrected distance visual acuity (UDVA), best corrected distance visual acuity (BCVA), refraction, keratometry, slit lamp biomicroscopy, indirect ophthalmoscopy, corneal topography, corneal astigmatism, endothelial cell count, and patient satisfaction. The follow-up period covered 12 months.

**Results:**

The mean uncorrected distance visual acuity (UDVA) and the best corrected visual acuity (BCVA) demonstrated statistically significant improvement after surgery in both groups. At the end of the follow-up period, the UCVA was statistically better for the patients with LRIs compared with those underwent ICL surgery alone. The LRIs group showed significant reduction in the mean topographic astigmatism from 1.48 ± 0.35 D preoperatively to 0.37 ± 0.14 D postoperatively (*P* < .0001) after one month. The control eyes did not show a statistically significant change (*P* > 0.05). The mean magnitude of the surgically induced astigmatism (SIA) read 1.10 ± 0.35 D,1.13 ± 0.34D,1.13 ± 0.34D,1.11 ± 0.35D by the end of the 1st, the ^3rd^, the ^6th^ and the 12th month postoperatively in LRIs group, which was slightly lower than the target-induced astigmatism (TIA). The difference in SIA between the LRI and the control group was statistically significant by the end of the 1st, the ^3rd^, the ^6th^ and the 12th month postoperatively (*P* < 0.001). The mean correction index (CI) was less than 1, which indicated undercorrection effect of limbal relaxing incision. No difference was observed in the postoperative endothelial cell count between the two groups. There was no intraoperative and postoperative ocular or systemic complication.

**Conclusion:**

Limbal relaxing incision is an effective method in reducing corneal astigmatism during implantable collamer lens surgery.

**Trial registration:**

The trial was retrospectively registered in 14 April 2017. (NO:ChiCTR-ONR-17011147).

**Electronic supplementary material:**

The online version of this article (doi:10.1186/s12886-017-0458-7) contains supplementary material, which is available to authorized users.

## Background

The Visian Implantable Collamer Lens (ICL) (STAAR Surgical Co.), a posterior chamber phakic intraocular lens (PIOL), has been reported to perform well for the correction of moderate to high ametropia [1–4]. At least 15% to 29% of population has 1.5 diopters (D) or more of corneal astigmatism at preoperative evaluation, the proporation of which may be higher in high myopia. Astigmatism(>0.75D) may cause asthenopia, blurring of vision, double images and decreased vision. Therefore, corneal astigmatism is an issue of major concern in modern refractive surgery.

Different techniques are available to correct astigmatism, such as arcuate keratotomy, limbal relaxing incisions, laser vision correction, and Toric Implantable Collamer Lens (TICL) implantation. One popular approach to correct corneal astigmatism simultaneously to cataract surgery is to treat pre-existing astigmatism by creating limbal relaxing incisions (LRIs) [5–7].The limbal relaxing incisions (LRIs) technique involves the placement of incisions corresponding to the steep meridian, resulting in corneal flattening and the reduction of astigmatic power. LRI is a safe and an inexpensive procedure, which is simple for experts to perform. It can effectively reduce astigmatism up to 3.0 D and result in a rapid visual rehabilitation. But there are few reports about reducing pre-existing corneal astigmatism by LRI in ICL surgery. Recently, Toric hyperopic ICLs(TICL) are currently available for clinical use. But the patients have to wait for a long time for a surgery. Besides, because of the expensive lens, the patients need to pay more in TICL surgery than in ICL surgery. But the residual of astigmatism and the misalignment of the TICL might be a critical error, for in the LRI group, even if there was misalignment of the ICL, a correctly performed LRI still provided correction, thus, the risk of a major critical error is decreased. Therefore, LRIs may be a simpler, safer, more economic and effective way to reduce some slight and moderate preexisting corneal astigmatism in ICL surgery. Our research was aimed to evaluate the clinical outcomes of LRIs in ICL implantation to moderate myopia with regular corneal astigmatism more than 0.50 diopter (D) and less than 3.00 D. Outcomes included visual and refractive results with specific attention to the astigmatism vector analysis. Follow-up last at least 12 months.

## Methods

The study was a prospective, analysis reviewing consecutive cases of corneal astigmatism that had either independent ICL surgery (control group) or combined with LRIs (LRIs group). The study was performed at the Department of Ophthalmology, Leshan People’s Hospital, Sichuan Province, China. This project was approved by the science and technology foundation of Sichuan Provincial Health and Family Planning Commission(NO.150065).

The study covered 85 eyes of 45 patients with high myopia and regular corneal astigmatism more than 0.50 diopter (D) and less than 3.00 D from January, 2015 to December, 2016. The study was approved by the local ethics committee and adhered to the tenets of the Declaration of Helsinki. Informed consent to use any clinical data for analysis and publication was obtained from all patients prior to surgery. These 62 eyes of 32 patients received ICL surgery combined with LRIs (limbal relaxing incision group) while the other 23 eyes of 13 patients received only ICL surgery (control group). All the surgeries were performed independently by one surgeon. Exclusion criteria included irregular corneal astigmatism, keratoconus or keratoconus suspect, current uveitis, marked corneal scarring, pannus, and pterygium in preoperative topography, unclear cornea, and with history of previous ocular surgery. Peripheral corneal thickness was greater than 660 μm and pupil size was greater than 5 mm (after mydriasis). Preoperatively and postoperatively, by the end of the 1st week of the 1st, the ^3rd^, the ^6th^ and the 12th month, all the patients underwent a complete ophthalmic examination that included uncorrected distance visual acuity (UDVA), corrected distance visual acuity (CDVA), refraction, keratometry, slit lamp biomicroscopy, indirect ophthalmoscopy, corneal topography, (Pentacam, Oculus, Germany), the horizontal white-to-white distance and anterior chamber depth, corneal astigmatism, intra-ocluar pressure(IOP) and endothelial cell density. Eendothelial cell density was determined using a noncontact specular microscope by one single operator (J.Y).(SP-8800, Konan, Nishinomiya, Japan). For all eyes, emmetropia was selected as the target refraction to reduce the preoperative refractive errors as much as possible. The UDVA and CDVA were examined using Snellen charts and converted to the logMAR scale for statistical analysis.

### Surgical procedures

**Fig. 1:**
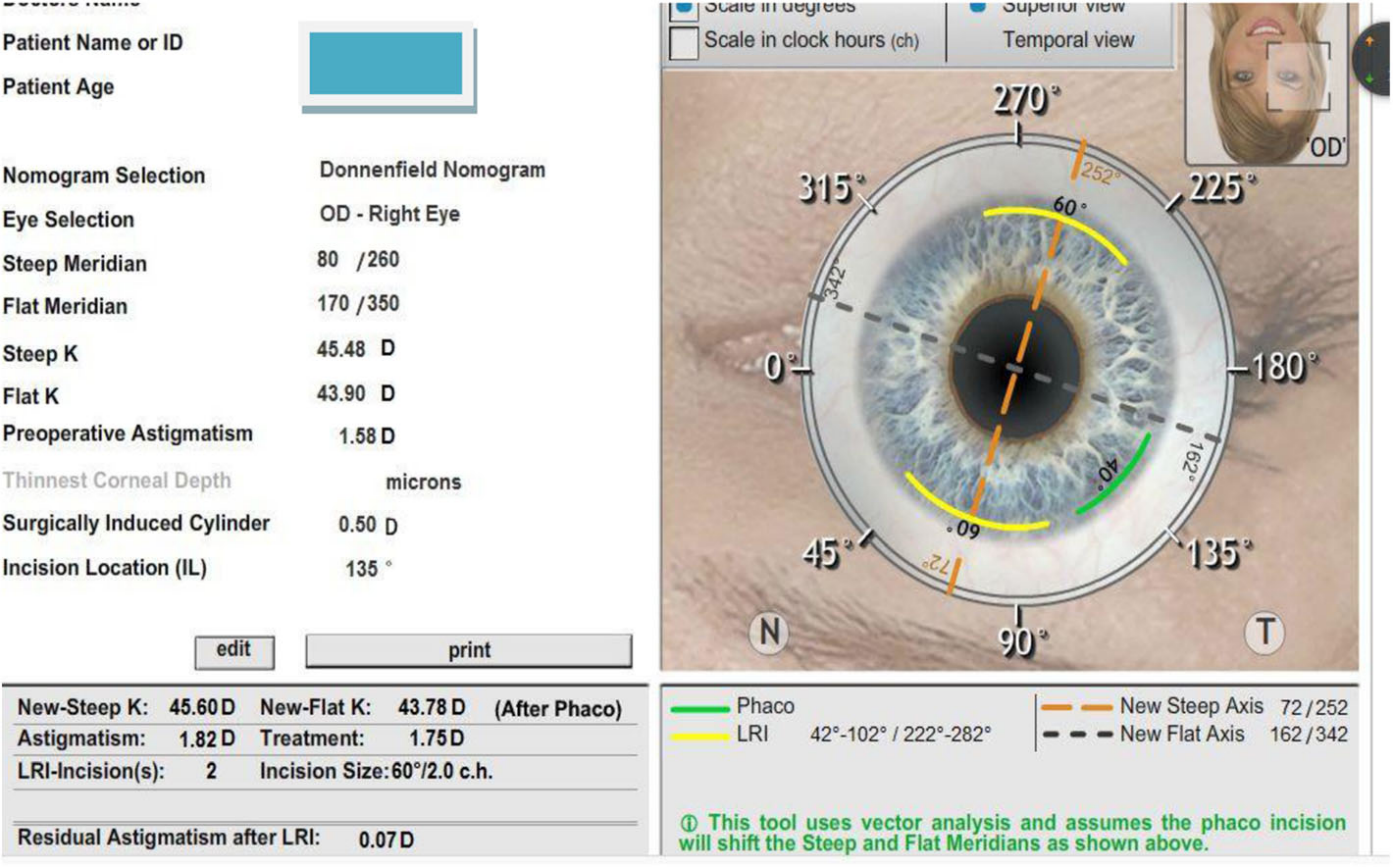
Example of LRI surgical planning (http://www.lricalculator.com - accessed may 1st, 2015)

Optimal axis locations for LRIs was determined by using an online software (Abbott Medical Optics, USA; available at http://www.lricalculator.com). Individual surgeon’s surgically induced astigmatism was considered as 0.5D.The online software uses Nichamin Age and Pachymetry Adjusted nomogram (available at http://www.lricalculator.com). Target refraction for all eyes was aimed at emmetropia. Figure 1 show the examples of LRI surgical planning.

Manual limbal markings at 0° and 180° were made for all eyes preoperatively with patients in sitting position at the slit lamp. A video-assisted eye tracking system was used to confirm accurate limbal markings intraoperatively. Surgeries were performed under topical anaesthesia in both groups. A Mendez-style ring was used to mark the steep meridians at the start of the surgery. In the LRIs group, the LRI incision was made before the commencement of Implantable Collamer Lens implantation. Based on the procedure described by Langerman [8], a vertical limbal relaxing wound was created with a guarded micrometer diamond blade by making a groove concentric to the limbus. The incision depth was set at 400-500 μm equal to approximately 85% of the peripheral corneal thickness After the LRI incision was made, ICL implantation was performed via a 3 mm temporal clear corneal incision. In the ICL group, all the patients did not perform LRIs.

Postoperatively, all the patients received regular follow-ups and a uniform postoperative drug regimen (combination of topical antibiotic and corticosteroids four times a day for 4 weeks in the operated eyes).

### Vector analysis

The surgical results of astigmatism were calculated, using the vector analysis method. The Alpins Goggins method [9] was used to measure surgically induced astigmatism (SIA), which is the vector of the real change achieved taking in consideration the amount and axis of astigmatism change induced by the surgery).SIA = Cpre –Cpost,which is alslo defined as the vector difference between the measured preoperative cylinder (Cpre) and the measured postoperative cylinder(Cpost). Target-induced astigmatism (TIA), which was the vector of intended change in cylinder. Additionally, correction index (CI) was calculated to evaluate the achieved power effect versus the targeted power, CI = |SIA|/|TIA| [10]. The ideal value would be close to 1; CI >1.0 D indicated overcorrection, while CI <1.0 D indicated undercorrection [11]. Calculations of Thibos vectors for refractive and corneal astigmatism were performed using Microsoft ExcelTM for MacIntosh spreadsheets.

### Statistical analysis

Statistical analysis was performed by using IBMTM SPSSTM for Microsoft WindowsTM software (version 20.0.0). Wilcoxon and Mann–Whitney nonparametric tests were performed for statistical analysis. The Wilcoxon test was used to analyze the difference between the preoperative evaluation and each postoperative evaluation within a group. The Mann–Whitney *U* test was used to determine differences between the limbal relaxing incision and control group preoperatively and postoperatively. A *P* value less than 0.05 was considered significant statistically.

## Results

A total of 85 eyes of 45 patients were included in this study. 62 eyes of 32 patients received ICL surgery combined with LRIs (limbal relaxing incision group) and 23 eyes of 13 patients received only ICL surgery (control group).

**Fig. 2:**
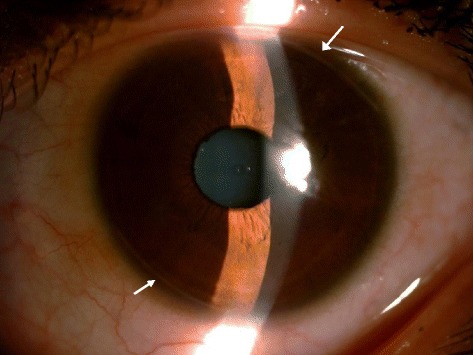
The picture of the LRI postoperatively(white arrows: location of the LRI)

No statistical difference was demonstrated between the two groups before surgery in terms of demographic characteristics, biometric data, UDVA, CDVA, refractive astigmatism, corneal astigmatism and topographic values (Table 1). There was no intraoperative and postoperative ocular or systemic complication. Figure 2 shows the picture of LRI postoperative.

**Table 1:** Comparison of baseline parameters between LRIs and control groups

	Groups		*P
Characteristics	LRIs	control	
Gender (female/male)	(25/7)	(10/3)	-
Age (years)	32.11 ± 10.39	31.09 ± 10.01	0.897
UDVA(logMAR)	0.03 ± 0.02	0.03 ± 0.04	0.424
CDVA(logMAR)	0.62 ± 0.24	0.63 ± 0.22	0.785
Refractive astigmatism (dioptre)	1.29 ± 0.56	1.46 ± 0.62	0.290
Corneal astigmatism (dioptre)	1.48 ± 0.35	1.43 ± 0.30	0.730
AL (mm)	28.31 ± 2.00	27.99 ± 2.51	0.536

*AL* Axial length, *LRI* Limbal relaxing incisions, *UDVA* uncorrected distance visual acuity, *CDVA* corrected distance visual acuity. *Mann-Whitney *U* test

### Visual outcomes

**Fig. 3:**
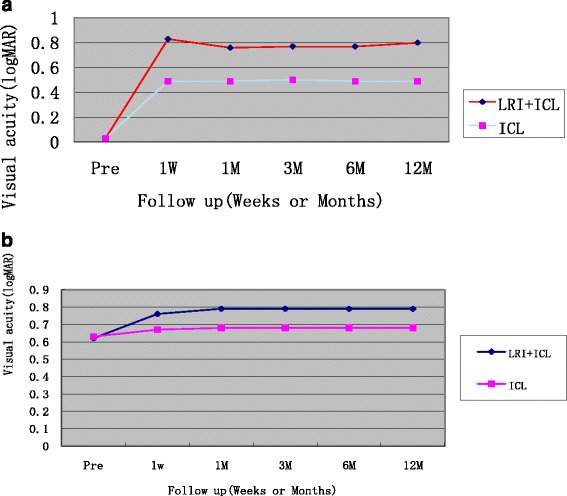
**a** UCVA is significantly better in the LRI + ICL group than in the LRI group over the follow-up period postoperatively (*P* < 0.01).Abbreviations: LRI, limbal relaxing incision; ICL, implantable collamer lens;Pre, preoperative; 1 W, 1 week postoperatively;1 M, 1 month postoperatively; 6 M, 6 months postoperatively;12 M,12 month postoperatively; logMAR, logarithm of minimum angle of resolution. **b** CDVA did not show statistically significant differences between the two groups(*P* > 0.05).Abbreviations: LRI, limbal relaxing incision; ICL, implantable collamer lens;Pre, preoperative; 1 W, 1 week postoperatively;1 M, 1 month postoperatively; 6 M, 6 months postoperatively;12 M,12 month postoperatively; logMAR, logarithm of minimum angle of resolution

The preoperative and postoperative UDVA and CDVA are shown in Fig. 3a and Fig. 3b. The postoperative UDVA and CDVA were significantly better than the baseline measurements for both groups (*p* ≤ 0.001). UDVA was statistically higher in the LRIS group compared with the control group (*P* < 0.05) over the follow-up period (Fig. 3a), while CDVA did not demonstrate statistically significant differences between the two groups (*P* > 0.05) (Fig. 3b).

### Topographic and Keratometric changes

**Fig. 4:**
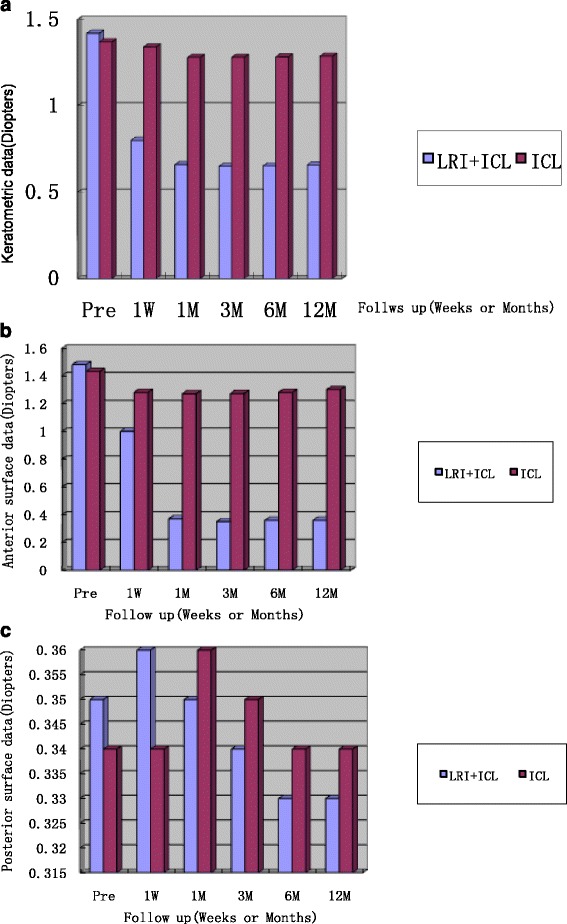
**a** Pre- and post-operative keratometric cylinder. Postoperative Keratometric cylinder was found to be significantly reduction in the LRI + ICL group over the follow-up period postoperatively (*p* < 0.05). The ICL group did not present a significant change in keratometric cylinder over the follow-up period postoperatively (*p* > 0.05). **b** Pre- and post-operative topographic anterior surface cylinder. Postoperative topographic anterior surface cylinder was found to be significantly reduction in the LRI + ICL group over the follow-up period postoperatively (*p* < 0.05). The ICL group did not present a significant change in topographic anterior surface cylinder over the follow-up period postoperatively (*p* > 0.05). **c** Pre- and post-operative topographic posterior surface cylinder. Postoperative topographic posterior surface cylinder was found to be no significantly variations in both groups over the follow-up period postoperatively (*p* > 0.05)

Anterior and posterior variations of the corneal surfaces were evaluated at 5 different points in time during the follow-up. They were 1w,1mo, 3mo,6mo and 12mo after surgery.At the end of the follow-up, a statistically significant reduction of the mean keratometric and topographic anterior cylinder were observed in the LRIs group. The control group did not present a significant change in keratometric and topographic astigmatism over the follow-up period. (Fig. 4a and Fig. 4b). The topographical posterior cornea surfaces did not demonstrate variations in both groups postoperatively (*P* > 0.05).(Fig. 4c). The mean spherical refraction was −11.50 ± 0.48D in the LRIs group and −12.75 ± 0.25D in the control group preoperatively. However, the mean spherical refraction was −0.12 ± 0.73D in the LRIs group and −0.13 ± 0.60D in the control group at 1 month postoperatively.

### Vector analysis of astigmatism

A statistically significant reduction in the mean topographic astigmatism was seen in the limbal relaxing incision eyes from 1.48 ± 0.35 D preoperatively to 0.37 ± 0.14 D after 1 month postoperatively (*P* < .0001). The control eyes did not show a statistically significant change in topographic astigmatism (preoperative astigmatism was 1.43 ± 0.30 to 1.27 ± 0.43 D at 1 month postoperatively (*P* > 0.05) as shown in Additional file 1: Fig. S5a and Fig. S5b).

Table 2 shows the postoperative changes in the vectors analyzed using the Alpins method in the limbal relaxing incision group. The mean magnitudes of the SIA were 1.10 ± 0.35 D,1.13 ± 0.34D,1.13 ± 0.34D,1.11 ± 0.35D at 1,3,6,12 month postoperatively, which were slightly lower than the TIA. The difference in SIA between the LRI and control groups was statistically significant at 1,3,6,12 month postoperatively (*P* < 0.001). The mean correction index (CI) was less than 1, which means undercorrection effect of limbal relaxing incision.

**Table 2:** Vector Analysis of postoperative Astigmatism for LRI and Control Groups

Time	Group		1 P
	LRI Group(Mean ± SD)	Control Group(Mean ± SD)	
TIA(D)	1.48 ± 0.35	-	-
SIA(D)			
1mo	1.10 ± 0.35	0.15 ± 0.52	*P* < 0.001
3mo	1.13 ± 0.34	0.16 ± 0.50	*P* < 0.001
6mo	1.13 ± 0.34	0.16 ± 0.51	*P* < 0.001
12mo	1.11 ± 0.35	0.13 ± 0.55	*P* < 0.001
2 P	*P* < 0.05		
CI(D)			
1mo	0.75 ± 0.11	-	-
3mo	0.76 ± 0.14	-	-
6mo	0.77 ± 0.10	-	-
12mo	0.77 ± 0.10	-	-
2	*P* < 0.05		

*LRI* limbal relaxing incision, *SD* standard deviation, *TIA* target induced astigmatism vector (the astigmatism changes [by magnitude and axis] the surgery was intended to be induced), *D* diopters, *SIA* surgically induced astigmatism vector (the amount and axis of astigmatism change induced by the surgery), *CI* correction index 2Wilcoxon Test; 1Mann-Whitney *U* test

### Endothelial cell count

The average loss of endothelial cells in both groups was regarded as having no statistical difference during follow- up period.

## Discussion

Astigmatism is one of the main ocular refractive defects which requires optical correction. To acquire good postoperative, uncorrected visual acuity, the astigmatism should be minimized. Refractive surgical procedures for eyes with astigmatism include incisional technique (arcuate keratotomy, corneal relaxing incisions, limbal relaxing incisions, or even on-axis phacoemulsification incision), laser vision technique (photorefractive keratectomy, LASIK, and toric IOLs), and a combination of techniques [12, 13]. Arcuate keratotomies or corneal relaxing incisions have limited predictability and often result in overcorrection, especially in eyes with low and moderate astigmatism. Currently, limbal relaxing incisions are the preferred technique to reduce preexisting astigmatism at the time of cataract surgery [11, 14–16]. Limbal relaxing incisions appear to have potential advantages over corneal relaxing incisions or arcuate keratotomy by causing less distortion and irregularity on corneal topographies and less variability in refraction because they are placed at the limbus. They can provide earlier stability in postoperative vision and may carry a lower risk of inducing glare and discomfort. In our study during the12 month follow-up, we did not find any intra or post-operative complication. In both groups, a significantly statistical increase in the BCVA was registered, while in the group treated with the LRI, a greater improvement of the UDVA was recorded, and the small residual astigmatism had no impacts quality of life postoperatively.

The results of the current study confirmed that LRIs are effective in reducing pre-existing corneal astigmatism of ≤3 D [17]The astigmatic reduction achieved in our study was comparable with the previously published reports. In our study, the limbal relaxing incision group showed significant reduction in astigmatism after postoperative (*P* < .0001), however, the control group did not show any significant difference in corneal astigmatism, which agree with the results of Alió et al. [18] and Elkady et al [19]. The mean reduction in topographic astigmatism was 1.11 D in the limbal relaxing incision group, which represents 75% reduction in preoperative topographic astigmatism. The variation in the reduction percentage may be due to difference in the number of cases, nomogram, number of limbal relaxing incisions, or the follow-up period.

In our study, there was no serious complication from limbal relaxing incisions with regard to infection, perforation, or even risk of denervation of the cornea with long incisions.

Although the LRI astigmatism correcting capability was affirmed, vector analysis with the SIA lower than TIA and CI < 1, which means undercorrection effect of limbal relaxing incision. As a method that is less dependent on the surgeons’ technical experience. The potential drawbacks of corneal incisions include risk of perforation, infection, unpredictability of results, creation of irregular astigmatism and disruption of the ocular surface [20–22]. However, it should be noted that there are certain negative aspects associated with LRI, such as that it involves a degree of uncertainty due to the difficulty of performing an equally relaxing incision, or that a relatively long amount of time is needed until stabilization of the correcting effect is acquired. As knowing that the regression of astigmatism can be as much as 1.00D [23], but in our study, we had found the regression of astigmatism would remain stable after 1 month postoperatively.

It is important to note that this present study did have some limitation, most of our patients undergoing ICL surgery is to deal with the army or recruitment enrollment physical examination, they usually hope to perform an operation immediately in order to restore a good eyesight in a short time. However,the patients have to wait for a long time to get the TICL from abroad. Therefore, these patients are more willing to choose ICL + LRI, instead of TICL. Also,there are small number of patients not willing to choose TICL due to economic reasons. After all, compared to ICL, they need to pay more in TICL. Due to the small number of cases, we did not have comparison in this manuscript. But now we are still in the cases of information collection, our further study will focuse on comparing LRI + ICL versus TICL, it will be more interesting.

## Conclusions

To summarise, LRI is effective in correcting corneal astigmatism during ICL surgery, while LRI tends to undercorrect astigmatism. Recently, the Toric hyperopic ICLs(TICL) currently represents an alternative method to the incisional technique for the slight and moderate astigmatism during ICL surgery. However, the misalignment of the TICL might be a critical error. Our further study will focuse on comparing LRI + ICL versus TICL. Moreover, we can address wider ranges of astigmatism, corneal aberration examination and other nomograms, and larger samples and longer follow-ups will be more desirable to confirm these results. Hope for the best result, economic power, surgeons’ preference, and the need for immediate visual recovery will decide the final choice.

## Additional file

Additional file 1: Figure S5a. Corneal astigmatism distribution in 2 groups preoperatively. **Figure S5b.** Corneal astigmatism distribution in 2 group 1 month postoperatively. (ZIP 20 kb)

## Abbreviations

BCVA: best corrected visual acuity
CDVA: corrected distance visual acuity
CI: correction index
D: diopters
ICL: implantable collamer lens
IOP: intra-ocluar pressure
LRIs: limbal relaxing incisions
PIOL: phakic intraocular lens
SIA: surgically induced astigmatism
TIA: target-induced astigmatism
TICL: Toric Implantable Collamer Lens
UDVA: uncorrected distance visual acuity
